# Correction: Dynamics of factors associated with neonatal death in Madagascar: A comparative analysis of the 2003, 2008, 2021 DHS

**DOI:** 10.1371/journal.pgph.0005424

**Published:** 2025-11-03

**Authors:** Sedera Radoniaina Rakotondrasoa, Kadari Cissé, Tieba Millogo, Hajalalaina Rabarisoa, Felix Alain, Seni Kouanda, Julio El-C. Rakotonirina

The Funding statement for this article is incorrect. The correct Funding statement is as follows: This project is part of the EDCTP2 programme supported by the European Union (grant number: CSA2020E-3132, PREP-EPID).
