# Supplementary material for: Dynamics of factors associated with neonatal death in Madagascar: A comparative analysis of the 2003, 2008, 2021 DHS
Source: PLOS Glob Public Health. 2025 Apr 29;5(4):e0003732. doi: 10.1371/journal.pgph.0003732 (PMC12040262; doi:10.1371/journal.pgph.0003732)
Supplement: Attachment — Submitted filename: Response to Reviewers.docx [file pgph.0003732.s002.docx]

**Manuscript ID: PGPH-D-24-02012**

**Manuscript Title: “Dynamics of factors associated with neonatal death in Madagascar: a comparative analysis of the 2003, 2008, 2021 DHS”**

**Journal: PLOS Global Public Health**

Dear Dr. Haghparast Bidgoli,

We are grateful for the opportunity to revise and resubmit our manuscript. We appreciate the thoughtful and constructive feedback provided by the reviewers, which has undoubtedly strengthened the quality and clarity of our work. We have carefully addressed each point raised by the reviewers and the editor, and we believe that the manuscript is now significantly improved.

Below, we provide a detailed point-by-point response to each comment. Changes made to the manuscript are highlighted in the revised manuscript with tracked changes, and a clean version is also submitted.

# A. Response to Editor's Comments:

## 1. Editor's Comment: Please add more explanation on how 'mother’s involvement in decision-making' is defined and categorized.

- Our Response: We appreciate the editor's request for clarification. In the revised manuscript, we have provided in **lines 196-201** a detailed explanation of how "mother's involvement in decision-making" was defined and categorized based on the DHS methodology. This variable was constructed using responses to questions regarding who usually decides on the respondent's healthcare, large household purchases, and visits to family or relatives. The categorization, follows a dichotomous "yes/no" approach: "yes" indicates that the mother makes decisions alone or jointly with her partner in at least one area, whereas "no" signifies that someone other than the mother is the decision-maker in all three areas.

## 2. Editor's Comment: Please add more explanation on how the wealth index was constructed and what is included in the index.

- Our Response: We have expanded the description of the wealth index in the Methods section (**lines** **187-195**). The wealth index is a composite measure of household living standards constructed using principal component analysis (PCA). It incorporates data on asset ownership (including television, radio, car, motorcycle, refrigerator, and telephone), dwelling characteristics (including flooring, roof, and wall materials, drinking water source, and toilet facilities), and other wealth-related indicators (including the number of rooms and agricultural land ownership). The resulting scores are standardized, summed per household, and then divided into population quintiles (poorest, poorer, middle, richer, and richest) to reflect the wealth distribution, following DHS methodology.

## 3. Editor's Comment: We do not publish any copyright or trademark symbols that usually accompany proprietary names, eg (R), (C), or TM (e.g. next to drug or reagent names). Please remove all instances of trademark/copyright symbols throughout the text, including Stata 17® on page 9 and 12.

- Our Response: We have carefully reviewed the manuscript and removed all trademark and copyright symbols, including those associated with Stata 17. (**lines** **223**)

## 4. Editor's Comment: Please provide an Author Summary. This should appear in your manuscript between the Abstract (if applicable) and the Introduction, and should be 150–200 words long. The aim should be to make your findings accessible to a wide audience that includes both scientists and non-scientists.

- Our Response: We have added an Author Summary that highlights the key findings and their implications for a broad audience, including both scientists and non-scientists. This summary can be found **in lines 39-53** of the revised manuscript.

## 5. Editor's Comment: Please review your reference list to ensure that it is complete and correct. If you have cited papers that have been retracted, please include the rationale for doing so in the manuscript text, or remove these references and replace them with relevant current references. Any changes to the reference list should be mentioned in the rebuttal letter that accompanies your revised manuscript. If you need to cite a retracted article, indicate the article’s retracted status in the References list and also include a citation and full reference for the retraction notice.

- Our Response: We have meticulously reviewed the reference list for completeness and accuracy. We confirm that no retracted papers are cited. We have updated a few references with their most recent versions where applicable (reference n.3, 4, 5, 28).

# B. Response to Reviewer 1's Comments:

## 6. Reviewer 1's Comment: "This study allows us to incorporate elements that demonstrate how social determinants of health affect the probability of dying of newborns in Madagascar in different locations on the island. As reported by the WHO Commission on Social Determinants of Health, health inequality contributes to poor health outcomes for poor and disadvantaged segments of society and is clearly visible in the results to be published."

Our Response: We sincerely thank Reviewer 1 for their positive feedback and recognition of the study's contribution to understanding the social determinants of health affecting neonatal mortality in Madagascar. We agree with the reviewer's observation that health inequalities contribute to poor health outcomes, particularly for disadvantaged segments of society. We have maintained this focus throughout the revised manuscript.

# C. Response to Reviewer 2's Comments:

## 7. Reviewer 2's Comment: "The article titled “Dynamics of factors associated with neonatal death in Madagascar: a comparative analysis of the 2003, 2008, 2021 DHS” presents a valuable and comprehensive analysis of neonatal mortality trends in Madagascar across nearly two decades."

Our Response: We thank Reviewer 2 for their comprehensive and insightful review, which has helped us improve the clarity and rigor of our manuscript. We appreciate the reviewer's positive assessment of our work and their acknowledgment of its significance for public health in Madagascar

## Introduction:

## 8. Reviewer 2's Comment: " The introduction effectively contextualizes neonatal mortality in Madagascar. However, I would suggest integrating lines 93 to 106 into the introduction, as they provide important contextual information that would enhance the reader’s understanding of the study's background from the outset. This section would benefit from a more fluid transition into the objectives and hypotheses of the study, aligning the study’s aims with the country’s public health challenges and goals. "

Our Response: Thank you for your thoughtful comment. While we understand the intent to improve narrative flow, we believe that the description of the study site (lines 93–106) aligns with Section 5 ("Setting") of the STROBE guidelines, which provides essential context for evaluating the study's generalizability.

The details about Madagascar’s socio-economic conditions and healthcare system are key to interpreting the results in their proper context. Understanding the geographic and temporal setting is crucial for assessing how environmental influences, health policies, and resources may affect the findings.

Given the length of the introduction, we aimed to keep it concise. We have kept the detailed description in the methodology to maintain clarity, separating the broader scientific rationale (in the introduction) from the specifics of the setting. To address your concern, we have streamlined the introduction **(lines 94–99)** to briefly mention Madagascar’s context and transition into the study’s objectives and hypotheses.

We hope this approach addresses your concerns while preserving the integrity of the manuscript.

## Methods :

## 9. Reviewer 2's Comment: " Data Collection Personnel and Process: It is not specified who collected the data (e.g., field researchers, healthcare professionals, etc.) and how this process was conducted. Additional information on the training and qualifications of those collecting data would add clarity, as well as details about quality control procedures during data collection. "

Our Response: We have added details about the data collection process, specifying that rigorously trained multidisciplinary teams consisting of a leader, an interviewer, and a health worker, collected the data. We have also included information on the training they received and the quality control measures implemented during data collection **(lines 150-160)**.

## 10. Reviewer 2's Comment: " Table 1 - High Response Rate: Achieving a 95% response rate is impressive. More detail on how this rate was maintained, particularly in hard-to-reach areas or among specific demographic groups, would be helpful in understanding any potential selection bias or limitations in the data. "

Our Response: We have added a sentence explaining that no areas with difficult access were excluded, and the organization of the data collections involved deploying the teams into the peripheral zones of the capital and the remote areas of the country before completing the central zones and the capital itself, which enabled the attainment and maintenance of high response rates. **(lines 153-156)**.

## 11. Reviewer 2's Comment: " Line 171 - Classification of Prenatal Visits: The categorization of prenatal visits (less than 3, 4 to 7, more than 8) appears somewhat ambiguous, as it is unclear where women who had exactly 8 visits are classified. A clearer breakdown or explanation would be beneficial.."

Our Response: We thank the reviewers for this observation. Indeed, women with exactly 8 visits are included in the "more than 8" category. Thus, we have modified the "more than 8" category to "8 or more" to address this confusion in the revised manuscript **(lines 211)**.

## 12. Reviewer 2's Comment : " Health Indicators: The study could be strengthened by including other important maternal and neonatal health indicators, such as rates of prematurity, intrauterine growth restriction, or maternal health conditions like gestational diabetes and HIV. These factors are often associated with neonatal mortality, and their absence may limit the study's scope. "

Our Response: While we acknowledge the importance of these indicators, our study was limited to the variables available in the DHS datasets. However, we have added a paragraph in the limitations section discussing the potential impact of these unmeasured variables and suggesting future research directions **(lines 550-554)**.

## 13. Reviewer 2's Comment: " There is a redundancy in mentioning “Stata 17®” on lines 183 and 250. This could be corrected for clarity."

Our Response: We have removed the redundant mention of Stata 17 and reformulated **line 223** to encompass both the utilization of Stata in data extraction and analysis.

## 14. Reviewer 2's Comment: " As a considerable portion of births may occur outside formal healthcare facilities, it would be relevant to know if data from traditional birth attendants were available or if there was information on births occurring in non-hospital settings. The distinction between neonatal deaths and deaths occurring in-utero prior to labor is often challenging to make. It would be helpful to clarify how these were differentiated in the dataset or if there are any limitations due to potential classification errors."

Our Response: We thank the reviewer for their valuable comments. We acknowledge that the consolidation of data regarding childbirth assistance into a dichotomous variable "assisted delivery by qualified healthcare personnel" (yes/no), despite the availability of information on traditional birth attendants, does not allow for the observation of their impact on neonatal mortality, a limitation we have detailed in the study's limitations section. Similarly, we recognize the potential for recall bias in maternally reported neonatal deaths, potentially leading to misclassification with stillbirths. While training addressed this issue, the absence of detailed medical records remains a limitation, also discussed in the limitations section. Future research should focus on the vital role of traditional birth attendants and employ more robust methods for accurate death classification **(lines 521-535; 556-563).**

**Results:**

## 15. Reviewer 2's Comment: " The results are well-presented and offer an insightful breakdown of various determinants of neonatal death. A few aspects could be further clarified: Definitions of Socioeconomic Indicators: Terms like “very poor households” require more precise definitions, as socio-economic indicators can vary greatly depending on geographic and cultural context. Clarifying the criteria used to define these categories would make it easier to interpret the results."

Our Response: We have added a clearer definition of the wealth index quintiles, including "very poor households" (now referred to as "poorest"), in the Methods section, as described in our response to the editor's comments **(lines 187-195)**.

## 16. Reviewer 2's Comment: " Decimal Places in Data: In some sections, results are presented with more than one decimal point, which can make them harder to read. Using only one decimal place throughout would enhance readability."

Our Response: We thank the reviewer for the suggestion regarding decimal places. We have revised the manuscript to use only one decimal place for percentages throughout, enhancing consistency and readability. However, we have retained two decimal places for Odds Ratios (ORs) and their 95% Confidence Intervals (CIs), as well as for the CIs of percentages, as this is standard practice to maintain precision in reporting these measures of association and their variability.

## 17. Reviewer 2's Comment: “Difficulty Accessing Health Center”: This factor is central to understanding neonatal mortality risks but would benefit from more detail. Is the difficulty due to financial constraints, geographical barriers, or limited health infrastructure? Specifying the components of this variable would enhance the study’s clarity."

Our Response: The variable initially named "Difficulty Accessing Health Center" was revised to "Geographic barriers to healthcare access" to accurately reflect its operationalization as the difficulty individuals face in accessing healthcare due to distance. Although factors such as cost, transportation, and decision-making power are acknowledged as potential barriers, they were excluded to maintain a specific focus on geographic challenges. This modification has been implemented throughout the text. The Methods section of the revised manuscript provides a detailed definition of this variable in lines 211-213.

## 18. Reviewer 2's Comment: " Formatting of Results: For instance, on lines 311–312, where the increase is given in both percentage and times, using a consistent format would improve clarity. Choosing either a percentage or fold-change format throughout the document would enhance readability. "

Our Response: We thank Reviewer 2 for their insightful comment regarding the formatting of results. To ensure consistency, we have revised the manuscript to express all changes using fold-change throughout. This involved converting percentages to fold-changes where necessary.

**Discussion :**

## 19. Reviewer 2's Comment : " The discussion is generally robust, providing valuable insights into how the results can inform health policy. However, a few additions could strengthen this section : Territorial Differences: The analysis of neonatal death prevalence by region and time period is very interesting, particularly the significant variability observed in urban settings between 2003 and 2008. However, the discussion does not address potential reasons for these shifts. Including possible explanations for regional differences, or highlighting any changes in healthcare access or policies, would provide valuable context."

Our Response: We have expanded the discussion to include potential explanations for the observed regional differences, such as variations in healthcare infrastructure, local health policies, and socioeconomic factors **(lines 441-454)**.

## 20. Reviewer 2's Comment : " The limitations regarding data quality could be reinforced. Given the nature of the data, quality is crucial and may influence the findings, potentially explaining the observed territorial differences. Additionally, improvements in data quality over time could impact the trends observed in the study. Furthermore, births occurring outside healthcare facilities, especially those assisted by traditional birth attendants, may be missed, leading to possible underreporting in certain areas. Moreover, neonatal deaths occurring shortly after birth are often undocumented or misclassified, which can result in inaccuracies and limit the precision of mortality estimates in the study"

Our Response: We have strengthened the discussion of data quality limitations, acknowledging potential biases and the impact of improvements in data collection over time on the observed trends **(lines 521-535)**. We have also added a discussion about births occurring outside healthcare facilities and the challenges of capturing neonatal deaths occurring shortly after birth.

# D. Conclusion:

We thank both reviewers and the editor for their valuable input, which has helped us refine our manuscript. We believe the revised manuscript is substantially improved and addresses all the concerns raised. We look forward to your positive response.

Sincerely,
